# Lactate saturation limits bicarbonate detection in hyperpolarized ^13^C‐pyruvate MRI of the brain

**DOI:** 10.1002/mrm.29290

**Published:** 2022-05-09

**Authors:** Nikolaj Bøgh, James T. Grist, Camilla W. Rasmussen, Lotte B. Bertelsen, Esben S. S. Hansen, Jakob U. Blicher, Damian J. Tyler, Christoffer Laustsen

**Affiliations:** ^1^ MR Research Center, Department of Clinical Medicine Aarhus University Aarhus Denmark; ^2^ Department of Physiology, Anatomy, and Genetics University of Oxford Oxford UK; ^3^ Oxford Center for Clinical Magnetic Resonance Research University of Oxford Oxford UK; ^4^ Department of Radiology Oxford University Hospitals Oxford UK; ^5^ Institute of Cancer and Genomic Sciences University of Birmingham Birmingham UK; ^6^ Center for Functionally Integrative Neuroscience Aarhus University Aarhus Denmark; ^7^ Department of Neurology Aalborg University Hospital Aalborg Denmark

**Keywords:** astrocyte neuron lactate shuttle, brain, hyperpolarized, magnetic resonance imaging, metabolism, pyruvate

## Abstract

**Purpose:**

To investigate the potential effects of [1‐^13^C]lactate RF saturation pulses on [^13^C]bicarbonate detection in hyperpolarized [1‐^13^C]pyruvate MRI of the brain.

**Methods:**

Thirteen healthy rats underwent MRI with hyperpolarized [1‐^13^C]pyruvate of either the brain (*n* = 8) or the kidneys, heart, and liver (*n* = 5). Dynamic, metabolite‐selective imaging was used in a cross‐over experiment in which [1‐^13^C]lactate was excited with either 0° or 90° flip angles. The [^13^C]bicarbonate SNR and apparent [1‐^13^C]pyruvate‐to‐[^13^C]bicarbonate conversion (*k*_PB_) were determined. Furthermore, simulations were performed to identify the SNR optimal flip‐angle scheme for detection of [1‐^13^C]lactate and [^13^C]bicarbonate.

**Results:**

In the brain, the [^13^C]bicarbonate SNR was 64% higher when [1‐^13^C]lactate was not excited (5.8 ± 1.5 vs 3.6 ± 1.3; 1.2 to 3.3–point increase; *p* = 0.0027). The apparent *k*_PB_ decreased 25% with [1‐^13^C]lactate saturation (0.0047 ± 0.0008 s^−1^ vs 0.0034 ± 0.0006 s^−1^; 95% confidence interval, 0.0006–0.0019 s^−1^ increase; *p* = 0.0049). These effects were not present in the kidneys, heart, or liver. Simulations suggest that the optimal [^13^C]bicarbonate SNR with a TR of 1 s in the brain is obtained with [^13^C]bicarbonate, [1‐^13^C]lactate, and [1‐^13^C]pyruvate flip angles of 60°, 15°, and 10°, respectively.

**Conclusions:**

Radiofrequency saturation pulses on [1‐^13^C]lactate limit [^13^C]bicarbonate detection in the brain specifically, which could be due to shuttling of lactate from astrocytes to neurons. Our results have important implications for experimental design in studies in which [^13^C]bicarbonate detection is warranted.

## 1 INTRODUCTION

Continuous and sufficient energy supply is vital for the viability and function of neurons.^1^ The brain's high energy consumption under normal physiological conditions is primarily satisfied by glucose oxidation. Acute disturbances in neuro‐energetic function are a hallmark of stroke or traumatic injury,^2^ while chronic disturbances are important contributors to degenerative, cancerous, and inflammatory diseases.^3^, ^4^, ^5^ One key site of metabolic imbalance is at the intersection of glycolysis and glucose oxidation, where pyruvate is metabolized to either acetyl‐CoA or lactate. New diagnostic tools capable of detecting imbalances in carbohydrate metabolism at this intersection would provide valuable information for clinicians and researchers alike.

Magnetic resonance imaging with hyperpolarized [1‐^13^C]pyruvate is an emerging clinical technology that enables imaging of pathway‐specific cerebral metabolism.^6^ After intravenous administration, the hyperpolarized [1‐^13^C]pyruvate, [1‐^13^C]lactate, and [^13^C]bicarbonate can be dynamically imaged, and conversion of [1‐^13^C]pyruvate to its metabolites can be quantified. The [1‐^13^C]lactate is formed by the lactate dehydrogenase. The [^13^C]bicarbonate is formed from decarboxylation of [1‐^13^C]pyruvate by the pyruvate dehydrogenase, followed by conversion of the resulting ^13^CO_2_ to [^13^C]bicarbonate by the carbonic anhydrase. As such, the [1‐^13^C]pyruvate represents a key metabolite, and its metabolic fate represents a readout of the energetic status of the cell. Imaging of [1‐^13^C]pyruvate‐to‐[1‐^13^C]lactate shows potential as a biomarker of mutational status and treatment response in brain cancers,^7^, ^8^, ^9^ progression of stroke,^10^, ^11^, ^12^ and inflammatory metabolism.^13^ However, [^13^C]bicarbonate, generated as [1‐^13^C]pyruvate enters mitochondrial metabolism, has to date received less attention, in part due to its low SNR.

The pools of lactate and pyruvate are in exchange through the lactate dehydrogenase. Even though back conversion from [1‐^13^C]lactate to [1‐^13^C]pyruvate is often neglected in hyperpolarized MRI, RF saturation of the [1‐^13^C]lactate frequency destroys the nonrecoverable magnetization that could have been transferred back to the bicarbonate pool through pyruvate. This transfer may occur to varying degrees between organs and physiological states, depending on the order and magnitude of the lactate dehydrogenase reaction, or it could even involve shuttling between cells. Regardless of the mechanism, a significant exchange from [1‐^13^C]lactate to [^13^C]bicarbonate combined with high flip‐angle excitation of [1‐^13^C]lactate will decrease the SNR of [^13^C]bicarbonate. However, detection of [1‐^13^C]pyruvate‐to‐[^13^C]bicarbonate metabolism is often warranted, as it may serve as a marker of mitochondrial metabolism, which is an important player in acute and choric neuronal injury.^5^, ^14^

The aim of this study was to demonstrate the effects of [1‐^13^C]lactate RF excitation on [^13^C]bicarbonate detection in MRI with hyperpolarized [1‐^13^C]pyruvate. We report that [1‐^13^C]lactate saturation decreases the SNR of [^13^C]bicarbonate as well as the apparent conversion of [1‐^13^C]pyruvate to [^13^C]bicarbonate in the brain specifically. Our findings inform the design of future studies of the brain where [^13^C]bicarbonate detection is warranted.

## 2 METHODS

### 2.1 Experimental animals

All animal experiments were approved by the Danish Animal Inspectorate. Male Sprague‐Dawley rats (*N* = 13, 8 weeks old, 240–310 g) from Taconic Biosciences (Denmark) were included. The rats were anaesthetized with 2.5%–3% sevoflurane in 2 L/min medical air. A tail vein catheter was placed for infusion of hyperpolarized [1‐^13^C]pyruvate, and normothermia was maintained using an MRI‐compatible small‐animal monitoring system (Small Animal Instruments). The animals underwent either single‐slice imaging of the brain (*n* = 8) or multislice imaging of the kidney, liver, and heart (*n* = 5).

### 2.2 Magnetic resonance imaging with hyperpolarized [1‐^13^C]pyruvate

Magnetic resonance imaging was performed on a 3T scanner (MR750; GE Healthcare) with a ^13^C/^1^H rat volume coil (RAPID Biomedical). The [1‐^13^C]pyruvic acid (127 mg; Cambridge Isotope Laboratories) was polarized in a 5T SPINLab (GE Healthcare) with AH111501 (15 mM; GE Healthcare) as the radical. After about 2 h, usually yielding polarization above 40%, the sample was dissoluted with heated water for injection and buffered to a final concentration of 125 mM [1‐^13^C]pyruvate. A volume of 1 ml was injected over 5 s through a tail vein catheter with each examination, and ^13^C‐imaging was initiated with the start of injection.

Anatomical images were acquired for reference. A T_2_‐weighted fast spin‐echo sequence (4600‐ms TR, 98.7 ms TE, 32 echo train length, 3‐mm slice thickness, 128 × 128 matrix for a 40‐mm FOV) was used for the brain, while a fast spin‐echo sequence was used for the body (8491‐ms TR, 8.3‐ms TE, 24 echo train length, 8‐mm slice thickness, 128 × 128 matrix for an 80‐mm FOV). A field map was acquired for assessment of B_0_ homogeneity (IDEAL IQ; 10.9‐ms TR, 4.1‐ms TE, 3 echo train length, 15‐mm slice thickness, 128 × 128 matrix). Hereafter, two ^13^C‐exams were performed in alternating order, only changing the [1‐^13^C]lactate flip angle from 90° to 0° or vice versa between them. A dynamic series of images were acquired using a gradient echo–type spiral sequence with spectral‐spatial metabolite‐selective excitation ([1‐^13^C]pyruvate/[^13^C]bicarbonate/[1‐^13^C]lactate flip angles = 8°/40°/90° or 8°/40°/0°). This previously described RF pulse was designed to minimize contamination between resonances.^15^ In the brain experiments, a single slice of 16 mm was acquired. In the body imaging experiments, three slices of 16 mm were acquired. In both experiments, 40 images were acquired with TRs of 500 ms or 650 ms, respectively, yielding a time resolution between two images of the same resonance of 1.5 s or 1.95 s. In brain imaging, the readout was a spiral with a 16 × 16 matrix, a 40‐mm FOV, and a 24‐ms readout. In body imaging, a spiral with 20 × 20 matrix, 80‐mm FOV, and 27‐ms readout was used. After imaging, a single localized spectrum was obtained to assess the prescription of the center frequency (5000‐Hz width, 2048 points, 30° flip angle). The two [1‐^13^C]pyruvate injections were performed about 30 min apart, and the person injecting was blinded to the experiment. The transmit gain was calibrated using a phantom with appropriate load and kept constant throughout the experiment. The carbon center frequency was extrapolated from the proton frequency and kept constant within the same animal.^16^

### 2.3 Processing and analyses of in vivo data

The in vivo ^13^C data were reconstructed and analyzed in *MATLAB* (MathWorks). After gridding, Fourier transformation, and denoising,^17^ the SNR of the area under the curve of each metabolite was measured over the entire organ (brain, kidney, liver, or heart with blood pool). For quantification of apparent [1‐^13^C]pyruvate‐to‐[^13^C]bicarbonate metabolism (*k*_PB_), a metabolic exchange model was fitted if SNR was > 3.^18^ The time to peak of [1‐^13^C]pyruvate (TTP) was derived as a simple measure of [1‐^13^C]pyruvate delivery and perfusion.^10^

### 2.4 Phantom experiments

To confirm the selectivity of the excitation pulse used for in vivo experiments, we measured its profile on a 50‐ml syringe filled with saline and 1:50 parts gadolinium‐based contrast agent with the rat volume coil. This experiment used Cartesian z‐encoding, a 16‐mm slice thickness, 7.5‐s TR, a 90° flip angle, and 256 excitations with a frequency shift of −4 Hz with each excitation. Additionally, a 1 M [1‐^13^C]lactate and a 1 M [^13^C]bicarbonate phantom were imaged with the center frequency shifted between the two. This experiment used the in vivo sequence with an 80‐mm^2^ FOV, a 1.5‐s TR, and 64 excitations. Finally, we assessed the B_1_^+^ profile of the coil using the Bloch‐Siegert approach.^19^ For this, a [1‐^13^C]pyruvate phantom with 1:50 parts gadolinium‐based contrast agent was placed in the middle of the coil and imaged using a soft pulse, 3‐s TR, three 16‐mm slices, a 60° flip angle, and 128 excitations.

### 2.5 Simulations

To evaluate the effect of different flip‐angle schemes on the detection of [^13^C]bicarbonate, the in vivo hyperpolarized data were used to inform simulations using equations previously described.^20^ An additional interaction to account for the conversion of [1‐^13^C]lactate to [^13^C]bicarbonate was included, with a term of *k*_LB_ introduced to simplify the system. This one‐way term assumed instantaneous conversion of [1‐^13^C]lactate to [^13^C]bicarbonate with no [1‐^13^C]pyruvate in between, as this intracellular pool of [1‐^13^C]pyruvate is much smaller and indistinguishable from the extracellular pool. The *k*_LB_ was represented by a decaying exponential modulated by the pool size of the [1‐^13^C]lactate pool, *k*_LB_, and the apparent T_1_ of [1‐^13^C]lactate. The simplified system can be seen in Figure 5A, and the equations governing the simulation are described in Equations (1) and (2):

(1) $$\mathrm{dB}/\mathrm{dt}=r_{B}*B+k_{\mathrm{LB}}*L$$

(2) $$\mathrm{dL}/\mathrm{dt}=k_{\mathrm{PL}}*P-r_{L}*L-k_{\mathrm{LB}}*L$$

where *r*_B_, and *r*_L_ are the relaxation rates of [^13^C]bicarbonate and [1‐^13^C]lactate under RF irradiation and T_1_ decay, respectively, and *k*_LB_ and *k*_PL_ are the apparent rates for the conversion of [1‐^13^C]lactate to [^13^C]bicarbonate and exchange of [1‐^13^C]pyruvate to [1‐^13^C]lactate, respectively. L, P, and B refer to the detectable [1‐^13^C]lactate pool, the detectable [1‐^13^C] pyruvate pool, and the detectable [^13^C]bicarbonate pool, respectively.

Each simulation assumed a TR of 1 s, 240 time steps, a *k*_PL_ of 0.012 s^−1^, and a *k*_LB_ of 0.001 s^−1^. The relaxation times, in the absence of RF irradiation, of [1‐^13^C]pyruvate, [1‐^13^C]lactate, and [^13^C]bicarbonate were assumed to be 35, 30, and 10 s, respectively. Simulations iterated over a 0°–90° flip angle on [1‐^13^C]pyruvate, [1‐^13^C]lactate, and [^13^C]bicarbonate, and the signal from each metabolite was summed over the time course. The SNR for each metabolite, for each simulation, was calculated using the summed time‐course signal and the SD of the noise in the final 10 points of the simulation.

### 2.6 Statistics

Statistical analyses and plotting were performed in R.^21^ Significance was tested using linear mixed‐effect models presented with effect estimates, confidence intervals (CIs), and *p*‐values.

## 3 RESULTS

### 3.1 Brain

**FIGURE 1:**
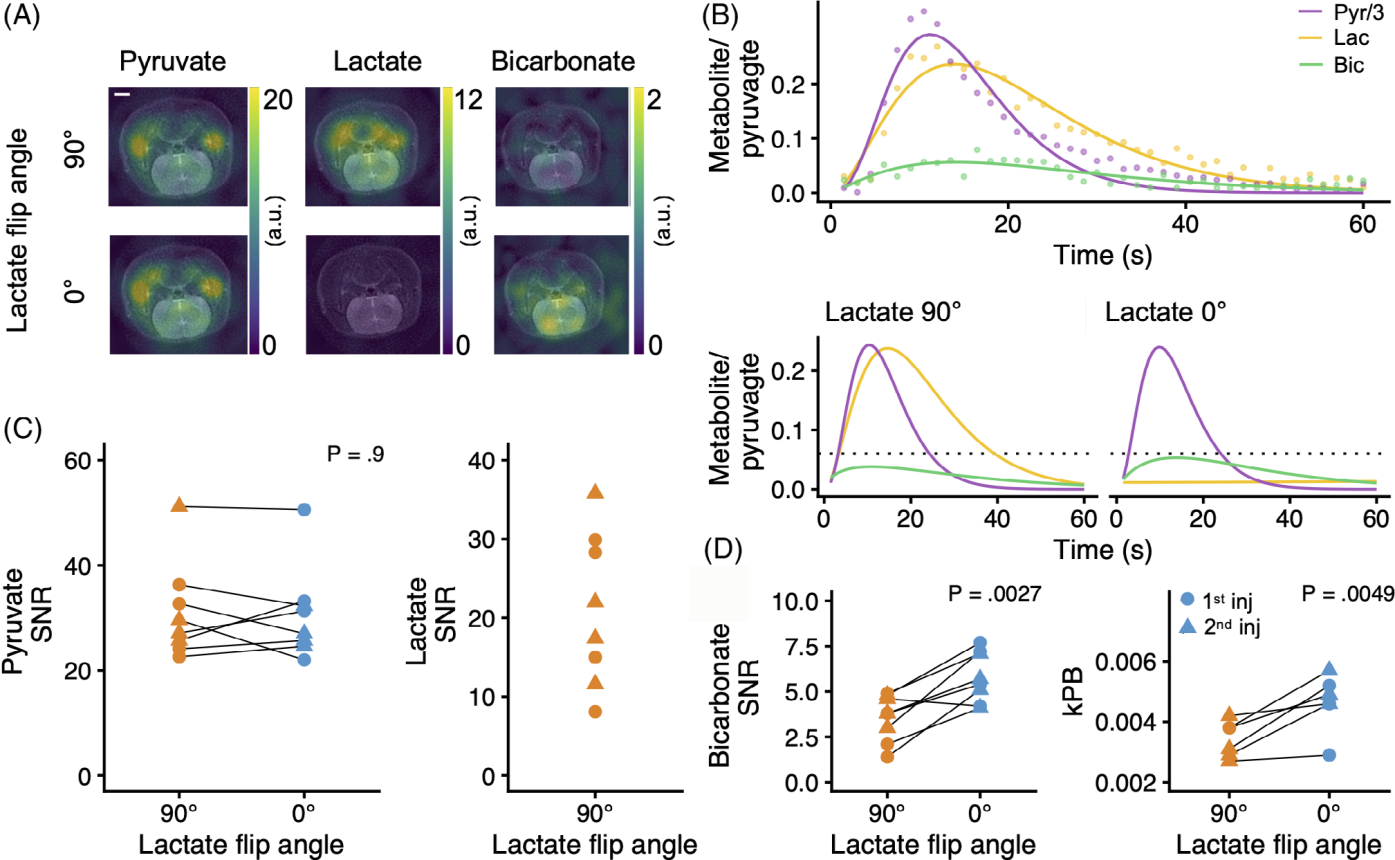
Magnetic resonance imaging with hyperpolarized [1‐^13^C]pyruvate was performed as a cross‐over experiment in healthy rats (*n* = 8) to evaluate the effects of [1‐^13^C]lactate excitation on the [^13^C]bicarbonate signal. A, Selectively exciting [1‐^13^C]lactate with a 90° pulse markedly decreased the [^13^C]bicarbonate signal (white scale bar is 2 mm). B, The data were quantified as the SNR and the apparent rate of [1‐^13^C]pyruvate‐to‐[^13^C]bicarbonate metabolism (*k*_PB_) determined from fitting of the time course (the top panel displays a single experiment with individual data points and the fit; the bottom panel displays all data aggregated). C, There was no difference in the [1‐^13^C]pyruvate SNR between a 0° or a 90° [1‐^13^C]lactate pulse, whereas the [1‐^13^C]lactate was only detectable with a 90° pulse. D, For [^13^C]bicarbonate, the 0° [1‐^13^C]lactate excitation led to larger SNR as well as higher *k*_PB_ (two data points were excluded due to insufficient SNR). This shows that the [^13^C]bicarbonate SNR is sensitive to [1‐^13^C]lactate excitation due to exchange from [1‐^13^C]lactate to [^13^C]bicarbonate. The curves in (B) were fitted as gamma‐variate models. Statistical significance was tested with linear mixed‐effects models

**FIGURE 2:**
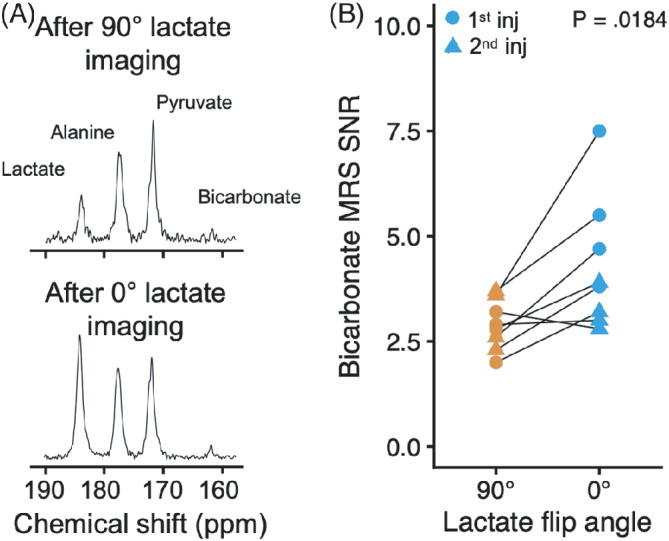
A, After imaging, a spectrum was acquired over the brain using a slice‐selective 30° pulse with a spectral bandwidth of 5000 Hz. B, This showed increased SNR of the [^13^C]bicarbonate peak at about 161.5 ppm after imaging with a 0° [1‐^13^C]lactate flip angle compared with [1‐^13^C]lactate saturation. The difference was tested with a linear mixed‐effects model

When comparing a 90° with a 0° [1‐^13^C]lactate excitation pulse in the brain (Figure 1), we found no difference in [1‐^13^C]pyruvate SNR, whereas [1‐^13^C]lactate was only detectable with the 90° pulse. The TTP of [1‐^13^C]pyruvate was 8.1 ± 2.5 s versus 8.5 ± 3.8 s between the two experiments (−0.4 difference; 95% CI, −1.8 to 1; *p* = 0.6). The SNR of [^13^C]bicarbonate was 5.8 ± 1.4 when [1‐^13^C]lactate was not excited versus 3.6 ± 1.3 when it was. This constitutes a significant 64% or 2.3‐point increase (95% CI, 1.2–3.3; *p* = 0.0027). When omitting the denoising step in postprocessing, we observed a similar [^13^C]bicarbonate SNR difference (4.1 ± 1.1 vs 2.3 ± 0.9; *p* = 0.008). Likewise, the *k*_PB_ was 35% higher with a 0° versus a 90° [1‐^13^C]lactate pulse (0.0047 ± 0.0008 s^−1^ vs 0.0034 ± 0.0006 s^−1^, 0.0012 s^−1^ increase; 95% CI, 0.0006–0.0019; *p* = 0.0049). The error of the *k*_PB_ was 0.0057 lower with no [1‐^13^C]lactate saturation (95% CI, −0.0023 to −0.0087; *p* = 0.011). Two data points from the 90° lactate flip‐angle experiment were excluded in the *k*_PB_ analysis due to insufficient SNR. In the spectra acquired after imaging (Figure 2), which were acquired with a soft‐pulse excitation, our results were similar with an increase in [^13^C]bicarbonate SNR from 4.3 ± 1.6 to 2.9 ± 0.6 with no [1‐^13^C]lactate saturation (48% or 1.4‐point increase; 95% CI, 0.45–2.4; *p* = 0.0184). The ^13^C‐examinations were prescribed with a mean frequency offset of −5.3 ± 13.9 Hz.

### 3.2 Heart, liver, and kidney

**FIGURE 3:**
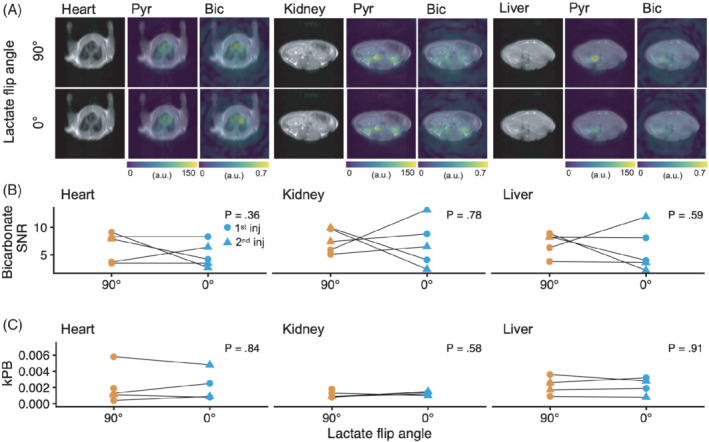
A, To assess the effects of [1‐^13^C]lactate excitation on the [^13^C]bicarbonate SNR in other organs than the brain, a subsequent series of cross‐over experiments was performed in healthy rats (*n* = 5). B, After injection of hyperpolarized [1‐^13^C]pyruvate, three slices of metabolite‐selective images (90° or 0° [1‐^13^C]lactate pulse) were acquired to evaluate the heart, kidneys, and liver separately. C, Following quantification, there was no difference in the [^13^C]bicarbonate SNR or apparent conversion of [1‐^13^C]pyruvate‐to‐[^13^C]bicarbonate (*k*_PB_ ). Statistical differences were assessed with linear mixed‐effects models

In a separate set of experiments, we evaluated the effects of [1‐^13^C]lactate excitation on the [^13^C]bicarbonate SNR outside the brain (Figure 3). We found no change in [^13^C]bicarbonate SNR when [1‐^13^C]lactate was not excited in either heart (−1.5; 95% CI, −4.5 to 1.6; *p* = 0.36), liver (−1.1; 95% CI, −5.0 to 2.8; *p* = 0.59), or kidney (−0.6; 95% CI, −4.7 to 3.5; *p* = 0.78). Furthermore, there was no change in the apparent *k*_PB_ in any of these organs.

### 3.3 Quality control and phantom experiments

**FIGURE 4:**
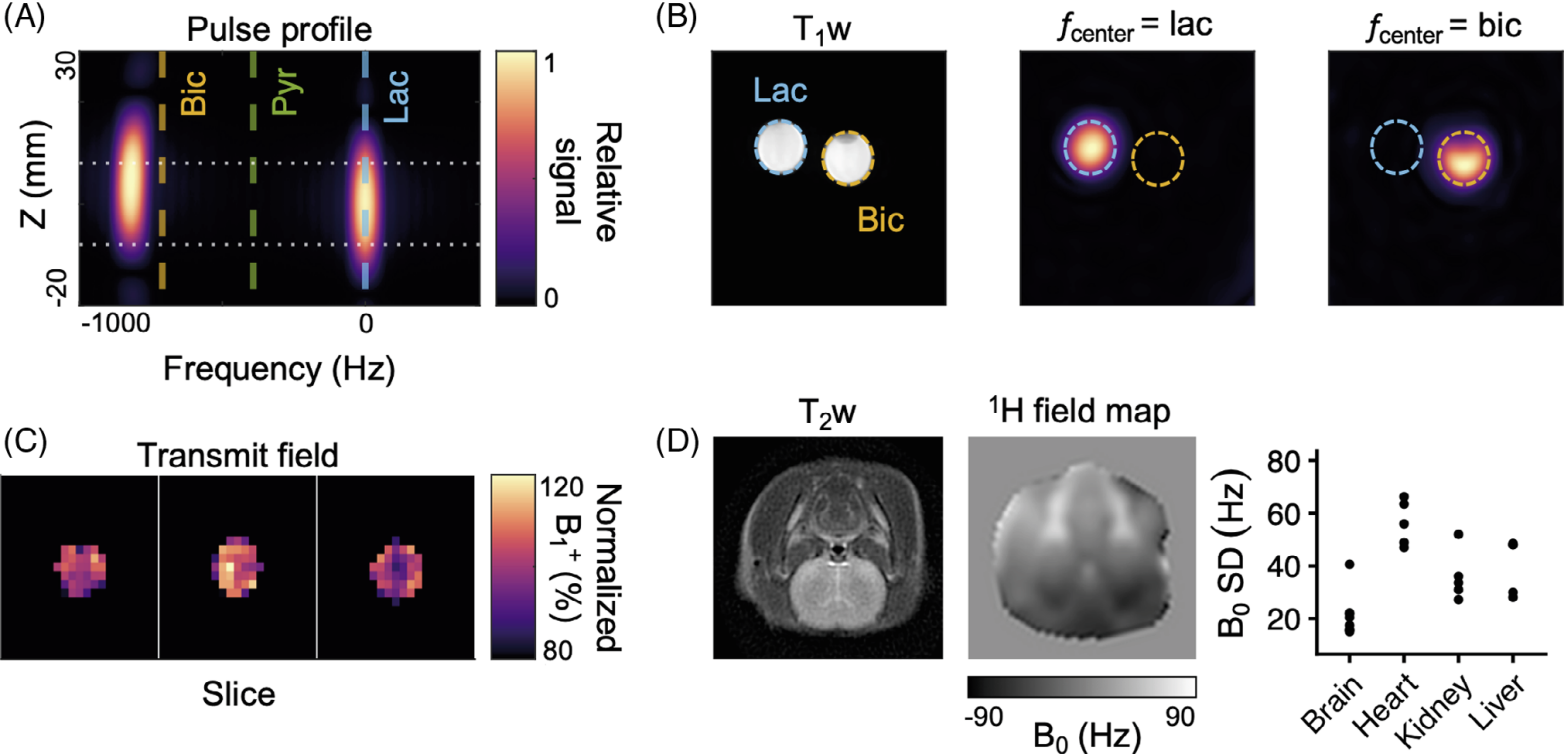
A series of phantom and quality experiments were performed, supporting the in vivo experiments. A, The measured profile of the excitation pulse was selective to the center frequency with minimal cross‐talk with other resonances (lactate is center frequency; other resonances marked with vertical lines; slice marked with horizontal lines). B, This was confirmed using the sequence from the in vivo experiments on 1 M [1‐^13^C]lactate (lac) and [^13^C]bicarbonate (bic) phantoms. Using the Bloch‐Siegert approach, a B_1_^+^ map was acquired of a [1‐^13^C]pyruvate phantom placed in the middle of the coil, mimicking the size and placement of a rat. Three 16‐mm slices were acquired. C, The map shows a relatively homogenous transmit field. D, Field maps show homogenous B_0_ across the brain, and more inhomogeneity in the body

To confirm the validity of the in vivo data, we performed a series of quality control experiments (Figure 4). We observed no off‐target excitation of [^13^C]bicarbonate when the center frequency was set to [1‐^13^C]lactate or vice versa. The normalized B_1_^+^ on a [1‐^13^C]pyruvate phantom was 100% ± 9.3%. In the in vivo experiments, the average SD of the B_0_ map across the brain was 21.1 Hz on the proton frequency, translating to about 5 Hz on the ^13^C‐frequency due to the lower gyromagnetic ratio.

### 3.4 Simulations on optimal flip angle

**FIGURE 5:**
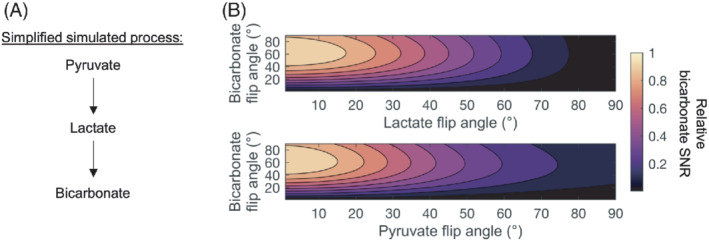
A, To inform the SNR optimal choices of flip angle in brain‐hyperpolarized MRI, a simplified simulation of [1‐^13^C]pyruvate‐to‐[^13^C]bicarbonate metabolism with [1‐^13^C]lactate as an intermediate was performed. The actual process is visualized in Figure 6B. Apparent rate constants of [1‐^13^C]pyruvate‐to‐[1‐^13^C]lactate (*k*_PL_) and [1‐^13^C]lactate‐to‐[^13^C]bicarbonate (*k*_LB_) were assumed to be 0.012 and 0.001 s^−1^, respectively. Longitudinal relaxation times were fixed to 35/30/10 s for [1‐^13^C]pyruvate/[1‐^13^C]lactate/[^13^C]bicarbonate, the fixed flip angle was set to 1°, and the TR to 1 s. The simulation indicates that the relative SNR of [^13^C]bicarbonate is highest with [1‐^13^C]pyruvate and [1‐^13^C]lactate flip angles of about 10° and a [^13^C]bicarbonate flip angle of 60°

Finally, we performed a series of simulations to inform the SNR optimal flip‐angle choice given the dependency of [^13^C]bicarbonate SNR on [1‐^13^C]lactate excitation (Figure 5). These show that the highest relative [^13^C]bicarbonate SNR is obtained with flip angles of 50°–70°, 5°–10°, and 5°–15° for [^13^C]bicarbonate, [1‐^13^C]pyruvate, and [1‐^13^C]lactate, respectively. Using these flip angles for [1‐^13^C]lactate and [1‐^13^C]pyruvate would decrease their respective SNRs from the simulated optimal, as a 20° [1‐^13^C]lactate flip angle gives a relative [1‐^13^C]lactate SNR of about 40%, whereas the same relative [1‐^13^C]pyruvate SNR is expected at a 10° flip angle.

## 4 DISCUSSION

These data show that RF saturation of [1‐^13^C]lactate limits detection of [^13^C]bicarbonate with hyperpolarized [1‐^13^C]pyruvate MRI of the brain. When [1‐^13^C]lactate was not saturated, [^13^C]bicarbonate SNR increased by 64%. This difference in SNR was not attributable to changes in [1‐^13^C]pyruvate delivery or polarization, as suggested by similar [1‐^13^C]pyruvate SNR and TTP. The experiments were performed in alternating order to avoid potential transient effects of the [1‐^13^C]pyruvate dose.^22^ Likewise, the robust MRS data confirm our imaging observations, and our phantom experiments confirm the spectral selectivity of the used pulse. Thus, the decrease in [^13^C]bicarbonate SNR was likely caused by saturation of [1‐^13^C]lactate, which is in exchange with [^13^C]bicarbonate.

In every cell, lactate is in exchange with pyruvate, as shown in previous saturation‐transfer experiments.^23^ Hereby, we would expect to see some saturation transfer between [1‐^13^C]lactate and [^13^C]bicarbonate. We were unable to observe this effect in the heart, kidneys, or liver. This was likely because we did not apply as efficient [1‐^13^C]lactate saturation pulses as in previous experiments.^23^ Instead, we observed that the decreased [^13^C]bicarbonate SNR with [1‐^13^C]lactate saturation was specific to the brain, suggesting that the exchange from lactate to bicarbonate is larger here than in the other organs.

**FIGURE 6:**
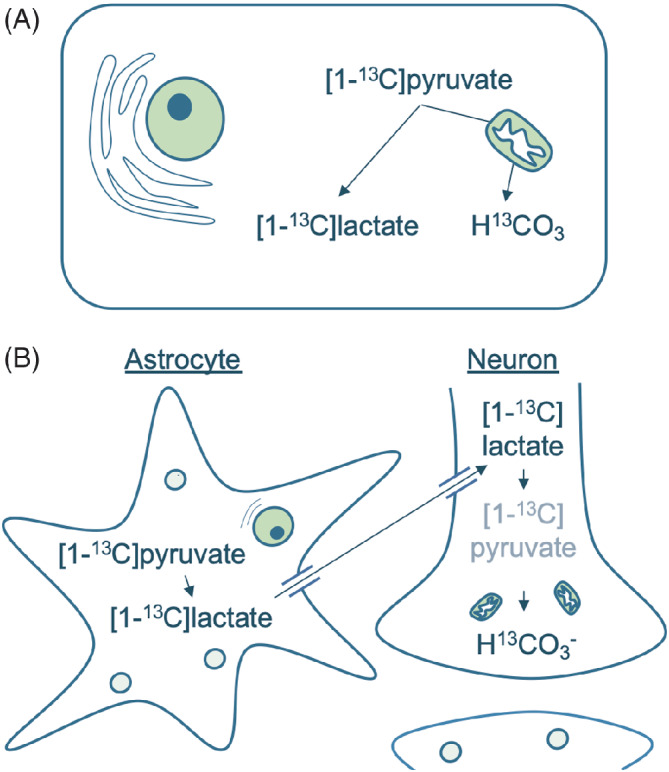
A, In MRI with hyperpolarized [1‐^13^C]pyruvate, the conversion of [1‐^13^C]pyruvate to [^13^C]bicarbonate is usually understood as a single‐cell process. B, However, in the brain, the actual process is likely more intricate. As suggested in the astrocyte‐neuron lactate shuttle hypothesis, the metabolism may be compartmentalized into glial [1‐^13^C]pyruvate‐to‐[1‐^13^C]lactate conversion and neuronal [1‐^13^C]lactate‐to‐[^13^C]bicarbonate conversion. This process involves several reactions and transports facilitated by the [1‐^13^C]lactate and [1‐^13^C]pyruvate dehydrogenases and monocarboxylate transporters 1, 2, and 4. The prolonged pathway leaves the ^13^C‐spins vulnerable to excitation at several points before finally being observable in [^13^C]bicarbonate

The difference in the observed [1‐^13^C]lactate‐to‐[^13^C]bicarbonate exchange between the brain and the heart, liver, or kidney may be explained by different [1‐^13^C]lactate handling in the brain. Magnetic resonance imaging with hyperpolarized [1‐^13^C]pyruvate is often conceptualized in a single‐cell model, where one cell metabolizes the [1‐^13^C]pyruvate (Figure 6). In actuality, brain metabolism appears to be compartmentalized, at least to some extent, as first hypothesized by Magistretti and Pellerin.^24^, ^25^ In their proposed astrocyte‐neuron lactate shuttle, lactate is produced in astrocytes and shipped to neurons for oxidation. The lactate is formed from pyruvate by the lactate dehydrogenase as the end‐product of glycolysis. It is then excreted from astrocytes by the monocarboxylate transporters 1 and 4, transported into neurons by the monocarboxylate transporter 2, and converted back to pyruvate, which is then oxidized. This shuttling of lactate is coupled with glutaminergic signaling and is thus driven by neuronal activity.^25^ If lactate is generated in astrocytes and bicarbonate in neurons, the ^13^C‐spins must exist in [1‐^13^C]lactate for a relatively long time before being observable in [^13^C]bicarbonate, and RF saturation applied to the [1‐^13^C]lactate frequency would hinder [^13^C]bicarbonate detection. However, the astrocyte‐neuron lactate shuttle hypothesis remains controversial.^25^ The difficulty of measuring real‐time metabolic fluxes in vivo has rendered modeling and in vitro work the methods of choice for both advocates and critics of the hypothesis.^25^ In vivo work with ^13^C‐labeled substrates has shown that neurons are able to metabolize lactate and that neuronal activity correlates with astrocytic glycolysis.^26^, ^27^, ^28^ Our data extend these findings by showing a brain‐specific RF saturation‐transfer effect from [1‐^13^C]lactate to [^13^C]bicarbonate during a real‐time 60‐s experiment. In addition to between‐cell shuttling, this could be explained by the brain, or just neurons, being net‐consumers lactate, leading to a larger within‐cell [1‐^13^C]lactate‐to‐[1‐^13^C]pyruvate flux or to back conversion from circulating hyperpolarized [1‐^13^C]lactate to [1‐^13^C]pyruvate. But this appears only to be the case during exercise, as the brain is a net producer of lactate under rest.^29^ Likewise, should the decrease of [^13^C]bicarbonate SNR be due to saturation of circulating hyperpolarized [1‐^13^C]lactate, we would expect to see a similar effect in the heart, which is also capable of lactate uptake. However, this was not observed. Thus, our observations are in agreement with a considerable shuttling of glial lactate to neurons for oxidation, even though differences in intracellular exchange from lactate to bicarbonate cannot be ruled out.

A compartmentalization of cerebral carbohydrate metabolism should be considered in the experimental design of hyperpolarized [1‐^13^C]pyruvate MRI. Recent sequence development in hyperpolarized [1‐^13^C]pyruvate MRI has popularized high flip‐angle excitation of [1‐^13^C]lactate with repetitive metabolite selective spectral‐spatial pulses. This is based on the understanding of [1‐^13^C]pyruvate‐to‐metabolite conversion as a single‐cell process with [1‐^13^C]lactate as an end metabolite. Furthermore, lactate saturation pulses may allow better and more robust estimation of enzyme kinetics than low flip‐angle excitations, particularly of reversible reactions.^11^, ^12^, ^15^, ^30^, ^31^ However, the data presented here show that saturating the [1‐^13^C]lactate frequency weighs the [^13^C]bicarbonate signal. If not taken into account, it leads to underestimated [1‐^13^C]pyruvate‐to‐[^13^C]bicarbonate conversion in the brain and causes lower [^13^C]bicarbonate SNR than warranted, such as for the detection of mitochondrial dysfunction accompanying neuronal injury.^14^ Of interest, CSI, which was widely used before dynamic spectral spatial approaches became prevalent,^32^ should be less prone to this effect due to a universally lower applied flip angle combined with an acquisition delay.

Our simulations should not be interpreted as a confirmation of a lactate shuttle effect. Rather, they are a pragmatic guidance on the optimal metabolite‐selective flip angles for detection of [^13^C]bicarbonate with acceptable [1‐^13^C]lactate and [1‐^13^C]pyruvate SNR given the observed dependence of the first on the latter. It is noted that the term *k*_LB_ is of interest, as both metabolites are primarily found in the intracellular space where they are formed. Any imaging of these metabolites gives an intracellular‐weighted image—in comparison to the largely extracellular‐weighted image of the [1‐^13^C]pyruvate pool. Therefore, it is challenging to separately simulate the *k*_LP_ and *k*_PB_ that constitute the k_LB_, as the intracellular pool of [1‐^13^C]pyruvate, which is metabolized to [1‐^13^C]lactate and [^13^C]bicarbonate, is not readily distinguishable from the large extracellular pool. Indeed, the intracellular pool of [1‐^13^C]pyruvate is small; therefore, assuming instantaneous conversion of [1‐^13^C]lactate to [^13^C]bicarbonate may be a valid assumption. Also, it is important to notice that the simulations assume total compartmentalization of astrocytic and neuronal metabolism. However, this is likely not the case,^25^ which may explain why the simulations overestimate the SNR gain compared with our experimental data. A flip angle of 40° was used for [^13^C]bicarbonate, which is suboptimal according to the simulations. A third cause for the discrepancy between the observed and simulated SNR gain may be the 1.5‐s time resolution, which could allow some flux of ^13^C‐spins through the lactate pool between two saturation pulses. Importantly, in the awake human brain, we expect the SNR penalty of [1‐^13^C]lactate saturation to be larger than suggested by our experimental data. This is due to the general effects of anesthesia on cerebral metabolism and hemodynamics,^33^ as well as the fact that neuronal activation stimulates lactate shuttling from glia cells to neurons and subsequent conversion from lactate to bicarbonate in neurons.^25^, ^31^, ^34^

In addition to sequence design, our results have implications for the kinetic modeling of apparent metabolism with hyperpolarized MRI. We observed an increase in the apparent *k*_PB_ when [1‐^13^C]lactate was not excited, even though no actual changes in metabolism would be expected. Thus, interpretation of *k*_PB_ in cerebral MRI with hyperpolarized [1‐^13^C]pyruvate should consider the [1‐^13^C]lactate excitation scheme. This is not corrected for in the current models.^35^ Previous efforts have considered more compartments in the kinetic modeling of hyperpolarized [1‐^13^C]pyruvate MRI,^36^ but, to our knowledge, no models have been developed for differentiating glial from neuronal metabolism. This should be the focus of future work, as lacking glial support of neuronal metabolism appears to be a player in neurodegenerative disease.^34^, ^37^ Such modeling would benefit from differentiation of intracellular and extracellular [1‐^13^C]lactate through DWI.^38^ Another approach could be to perform experiments with varying degrees of [1‐^13^C]lactate excitation. Of particular interest, future work should compare hyperpolarized [1‐^13^C]lactate, which is a separate ^13^C‐probe, to hyperpolarized [1‐^13^C]pyruvate, as the former may be more weighted toward neuronal metabolism as it would be directly metabolized by them.^39^

## 5 CONCLUSIONS

We report that [1‐^13^C]lactate saturation pulses decreases [^13^C]bicarbonate SNR in the brain, but not in the kidney, liver, or heart. This should be considered by applying lower [1‐^13^C]lactate flip angles when [^13^C]bicarbonate detection is warranted in brain hyperpolarized MRI. Furthermore, our findings have implications for interpretation of current kinetic models and indicate that brain‐specific models may need to be developed. One plausible explanation of our observations is lactate shuttling in the brain; therefore, we provide suggestions for future studies that may confirm and expand our findings toward differentiation of neuronal and glial metabolism using MRI hyperpolarized [1‐^13^C]pyruvate.
